# Cerebral Endothelial CXCR2 Promotes Neutrophil Transmigration into Central Nervous System in LPS-Induced Septic Encephalopathy

**DOI:** 10.3390/biomedicines12071536

**Published:** 2024-07-11

**Authors:** Fengjiao Wu, Yuhong Han, Qianqian Xiong, Haitao Tang, Jing Shi, Qingqing Yang, Xuemeng Li, Haoxuan Jia, Jun Qian, Yishu Dong, Tuantuan Li, Yong Gao, Zhongqing Qian, Hongtao Wang, Ting Wang

**Affiliations:** 1 Anhui Provincial Key Laboratory of Immunology in Chronic Diseases, Anhui Provincial Key Laboratory of Infection and Immunology, Department of Laboratory Medicine, Bengbu Medical University, Bengbu 233030, China; 2 Department of Clinical Laboratory, The Second People’s Hospital of Fuyang City, Fuyang 236015, China; 3 Department of Clinical Laboratory, Nanjing Meishan Hospital, Nanjing 210041, China; 4 Center for Translational Science, Florida International University, 11350 SW Village Parkway, Port St. Lucie, FL 34987, USA; 5 Department of Internal Medicine, University of Arizona, Phoenix, AZ 85004, USA

**Keywords:** BBB, CXCR2, endothelial barrier, septic encephalopathy

## Abstract

Septic encephalopathy (SE) represents a severe inflammatory syndrome linked to elevated septic mortality rates, lacking specific therapeutic interventions, and often resulting in enduring neurological sequelae. The present investigation endeavors to elucidate the involvement of C-X-C Motif Chemokine Receptor 2 (CXCR2) in the pathogenesis of SE and to explore the potential of CXCR2 modulation as a therapeutic avenue for SE. Employing a murine SE model induced by lipopolysaccharide (LPS) administration, CXCR2 knockout mice and the CXCR2 inhibitor SB225002 were utilized to assess neutrophil recruitment, endothelial integrity, and transendothelial migration. Our findings substantiate that either CXCR2 deficiency or its inhibition curtails neutrophil recruitment without impacting their adhesion to cerebral endothelial cells. This phenomenon is contingent upon endothelial CXCR2 expression rather than CXCR2’s presence on neutrophils. Furthermore, the CXCR2 blockade preserves the integrity of tight junction protein ZO-1 and mitigates F-actin stress fiber formation in cerebral endothelial cells following septic challenge. Mechanistically, CXCL1-mediated CXCR2 activation triggers cerebral endothelial actin contraction via Rho signaling, thereby facilitating neutrophil transmigration in SE. These observations advocate for the potential therapeutic efficacy of CXCR2 inhibition in managing SE.

## 1. Introduction

Sepsis is an organ dysfunction caused by an uncontrolled systemic inflammation in response to various infections [1]. The brain is one of the first organs to suffer from injuries caused by sepsis, which resulted in up to 70% of septic patients developing a diffuse brain dysfunction named “septic encephalopathy (SE)” or sepsis-associated encephalopathy (SAE) [2]. SE is associated with higher septic mortality without available specific therapy, and often leads to poorer long-term outcomes, due to impaired brain function, including altered mental status, reduced consciousness, cognitive impairment, as well as other neurological symptoms [3]. Despite the high incidence and clinical mortality, the pathomechanisms of SE are only partially elucidated, and there are no established specific therapeutic options for SE. Therefore, there is an urgent need to elucidate the complex pathophysiology and molecular mechanisms in acute and chronic SE to develop novel and specific concepts for preventing and treating SE.

SE is characterized by inflammation caused by endothelial and glial activation, blood–brain barrier (BBB) disruption, tissue hypoxia, neurotransmitter imbalance, and neuronal loss [4]. Notably, BBB disruption is confirmed to be a major contributor to the onset of SE. The BBB, formed mainly by cerebral microvascular endothelial cells, perivascular astrocytes, and pericytes, is a highly sophisticated and dynamic microvascular barrier that protects the brain parenchyma from the entry of pathogens, toxins, detrimental cells, and other macromolecules, and is a vital structure that helps to maintain brain homeostasis [5]. Dysfunction of the BBB is associated with various neurological disorders, highlighting its significance in brain health and function.

In fact, the cerebral vascular endothelium is the first barrier to the brain to interact with inflammatory mediators in blood circulation. During a septic challenge, endothelial integrity is broken, resulting in an enormous influx of circulating injurious bacteria debris, inflammatory immune cells, and other inflammatory mediators into the host tissue, including the brain [6]. During the development of SE, polymorphonuclear neutrophils (PMNs) are the most abundantly recruited proinflammatory immune cells, aiming to eliminate the local infection and healing of the injury [7]. Once the migration of neutrophils into the local inflammation occurs, however, their otherwise short half-life in tissue is extended by inflammatory mediators such as Granulocyte–Macrophage Colony-Stimulating Factor (GM-CSF), chemokines, and other chemoattractant molecules [8]. Current evidence suggests that neutrophil recruitment can be both a protective event by facilitating an efficient resolution to the clearance of systemic microbial infections, and also a tissue-damaging event by sustaining neutrophil activation and producing inflammatory mediators through delayed apoptosis, which contributes to nonspecific tissue injury in sepsis [9,10]. Thus, it is essential to reveal the exact mechanisms underlying the cerebral endothelial activation and neutrophil recruitment into the brain in SE, in order to identify more effective strategies to interrupt these deleterious responses.

C-X-C Motif Chemokine Receptor 2 (CXCR2) is a G protein-coupled receptor with the strong ligand chemokine CXCL1 (the functional IL-8 homologue in humans). CXCR2 expressed on neutrophils has been reported to play a critical role in the recruitment of neutrophils in preclinical models of arthritis, allergy, respiratory disease, and ulcerative colitis [11]. The activation of Toll-like receptors (TLRs) in neutrophils downregulates CXCR2 expression and impairs neutrophil migration in sepsis [12]. However, CXCR2 deficiency caused the failure to recruit neutrophils without affecting the neutrophil adhesion on the surface of cerebral endothelium in a murine SE model [13], suggesting that other chemokines may compensate for the chemotactic effect of CXCR2. Recent evidence and our previous results reveal that the regulation of endothelial CXCR2 expression is important for maintaining endothelial integrity [14,15]. Furthermore, IL-8 levels were increased in the CSF and brains of SE patients, resulting in a higher accuracy in the diagnosis of SE [16,17].

The infiltration of neutrophils from circulation to the brain is a coordinated multi-stage process, including capture, rolling, firm adhesion, and transendothelial migration [18,19]. In the transendothelial migration, the tight junctions of the BBB composed of proteins, including zonula occludens-1 (ZO-1) play a key role. ZO-1 binds to the actin cytoskeleton, and its localization provides valuable information on the status of BBB integrity and permeability [20]. F-actin stress fiber formation significantly influences the rate and size of the inter-endothelial cell gap that forms as cells retract from their borders, which subsequently regulates the transendothelial migration step of neutrophils [21]. CXCR2 is reported to mediate IL-8-triggered paraendothelial barrier breakdown in multiple sclerosis [14]. But, mechanisms underlying CXCR2 that promote the step of neutrophil transendothelial migration in SE through the regulation of ZO-1 distribution and F-actin stress fiber formation remain to be illustrated.

In this study, we confirmed that CXCR2^−/−^ mice fail to recruit neutrophils to the brain in a murine SE model from LPS-induced sepsis, but there is a similar level of adhesive neutrophils on the surface of cerebral endothelial cells, which is also verified by CXCR2 inhibitor SB225002. Furthermore, we determined that a CXCR2 deficiency and CXCR2 inhibitor SB225002 both attenuated the disruption of ZO-1 redistribution and F-actin stress fiber formation in primary cerebral endothelial cells. Finally, we found that CXCL1-triggered CXCR2 activation phosphorylated Myosin Light Chain 2 (MLC2) through Rho signaling, which subsequently induced cerebral endothelial actin contraction and the final process of neutrophil transmigration in SE, suggesting the potential clinical treatment with CXCR2 inhibitor SB225002 in SE.

## 2. Materials and Methods

### 2.1. Animals and Chemicals

C57BL/6J male mice (8–10 weeks) used as wild-type controls were purchased from Animal Institute (GemPharmatech, Nanjing, China). CXCR2^−/−^ mice (on the C57BL/6J background) were purchased from the Jackson Laboratory (Bar Harbor, ME, USA). All mice were maintained under environmentally controlled conditions (ambient temperature, 22 ± 2 °C; humidity 40%) in a pathogen-free facility with a 12 h light/dark cycle and had access to water and food ad libitum. All experimental procedures were performed in strict accordance with the Animal Care and Use Committee of Bengbu Medical College (#2018066). The murine septic encephalopathy model was induced by intraperitoneal injection of LPS (10 mg/kg, Invitrogen, Carlsbad, CA, USA) in 200 mL of PBS. Control mice received the injection of PBS with the same volume. To block CXCR2 in vivo, CXCR2 antagonist SB225002 (10 mg/kg, Cayman, Ann Arbor, MI, USA) was intraperitoneally injected 0.5 h prior to LPS injection. Vehicle control mice received injections of the same volume of 0.1% DMSO (RNBH9958, Sigma, St. Louis, MO, USA) in PBS.

### 2.2. Cell Culture

bEND.3 cells (primary mouse brain endothelial cells) [22] were obtained from Dr. Mingshun Zhang’s lab, located in Nanjing Medical University, China. Cells were cultured in high-glucose DMEM (9122470, Gibco, Carlsbad, CA, USA) medium with 10% FBS (42A0378K, Gibco) at 37 °C and 5% CO_2_ in a humidified incubator. To block CXCR2 in bEND.3 cells, cells were pretreated for 30 min with the 20 nM SB225002 (13336, Cayman). To block Rho kinase, cells were pretreated for 30 min with 2.5 nM of Y-27632 (10005583, Cayman).

### 2.3. Endothelial Transwell Permeability Assay

bEND.3 endothelial permeability was evaluated using the Endothelial Transwell Permeability Assay Kit (CB6929, Cell Biologics, Chicago, IL, USA). All experimental procedures followed the manufacturer’s protocol. Briefly, bEND.3 cells were seeded on the transwell insert membrane and cultured in an endothelial growth medium until confluency. Cells were then starved in a serum-free medium for 4 h, and reagents (recombinant CXCL1, SB225002, and streptavidin-HRP) were added into the top insert chambers. After the stimulation, media samples (20 μL) from the lower chamber were collected and the levels of leaked streptavidin-HRP were determined by TMB substrate. Relative endothelial permeability was calculated using the relative levels of leaked HRP through the endothelial monolayer using the method suggested by the kit.

### 2.4. Immunohistochemistry

After euthanization, the mice were quickly transcardially perfused with ice-cold 4% paraformaldehyde solution (Sigma). Mouse brain blocks were embedded in paraffin after fixation and sliced into 4 μm sections. For the detection of infiltrating neutrophils, infiltrating neutrophils were stained using a Naphthol AS-D Chloroacetate Specific Esterase Kit (90C2, Sigma). Fields of typical view at a primary magnification of ×400 in the cortex of every brain section were selected.

### 2.5. Western Blot and Antibodies

After treatment, cells were harvested and lysed by cell lysis buffer (P0013B, Beyotime, Shanghai, China) containing protease inhibitor (P1005, Beyotime) and phosphatase inhibitors (BL615A, Biosharp, Guangzhou, Guangdong, China). Protein concentrations in cell lysates were determined with a bicinchoninic acid (BCA) kit (P0010, Beyotime). Extracted cellular protein (50 μg) was mixed with SDS-PAGE sample loading buffer (P0015, Beyotime), boiled for 5 min, and then loaded onto SDS-PAGE gel. After the electrophoresis separation, proteins in gels were transferred to PVDF membranes (0.22 μm, Millipore, Burlington, MA, USA) at 200 mA for 2 h. The membranes were incubated in block buffer (5% BSA in TBST buffer, Biosharp) for 1 h, then incubated with the indicated primary antibodies in block buffer at 4 °C overnight. After washing three times in TBST for 10 min each, the membranes were incubated correspondingly with horseradish peroxidase (HRP)-conjugated goat anti-rabbit IgG antibodies (1:5000, A0208, Beyotime) at room temperature for 2 h. After washing three times in TBST for 10 min each, the protein bands were visualized via ECL buffer (Pierce, Waltham, MA, USA) in a luminescent image analyzer (ImageQuant LAS 4000 mini) and quantified by densitometry analysis (Image J version 1.54b). The antibodies were obtained as follows: anti-β-actin rabbit antibody (1:1000, GB11001, Solarbio, Beijing, China), anti-GAPDH rabbit polyclonal antibody (1:1000, 10494-1-AP, Proteintech, Rosemont, IL, USA), anti-VCAM-1 polyclonal antibody (1:1000, ab134047, Abcam, Cambridge, UK), anti-CXCR2 polyclonal antibody (1:1000, BOS6400BP3045, Boster Bio, Pleasanton, CA, USA), anti-mouse albumin antibody (1:500, ab19194, Abcam), MLC2 rabbit mAb (1:1000, 3672, Cell Signaling, Danvers, MA, USA), Phospho-MLC2 rabbit mAb (1:1000, 3671, Cell Signaling).

### 2.6. Immunofluorescence Staining

Primary cerebral microvascular endothelial cells were cultured on gelatin-coated glass coverslips until confluency and starved for 4 h with serum-free medium. Cells were then treated with CXCL1 (200 ng/mL, OW0319011, BD Biosciences, Franklin Lakes, NJ, USA), SB225002 (20 nM), and/or Y-27632 (2.5 nM, Sigma) for 100 min. After stimulation, cells were washed with cold PBS, fixed with 4% paraformaldehyde, and permeabilized with 0.2% Triton X-100. After being blocked with 5% normal goat serum in TBST, the cells were incubated with mouse monoclonal antibody ZO-1 (1:50, Invitrogen) at 4 °C overnight. After washing three times with cold TBST (5 min each), the cells were incubated with anti-mouse IgG Alexa Fluor 488 (Abcam) for 2 h at room temperature. The nuclei were stained with 4′,6-diamidino-2-phenylindole (DAPI, Abcam) for 5 min at room temperature. For F-actin staining, cells were placed with fluorescein phalloidin (1:20,000, MW:1177.27, Invitrogen) for 60 min at room temperature. Fluorescence was observed with a ZEISS inverted fluorescence microscope.

### 2.7. Flow Cytometry

Flow cytometric analysis of single-cell suspensions prepared from peripheral blood was performed on a Beckman CytoFlex (Cytek Biosciences, Fremont, CA, USA). Antibody clones used for staining were specific for Ly-6G (2376085, Invitrogen), CXCR2 (242216, R&D systems, Minneapolis, MN, USA), and IgG2A APC-conjugated antibody (F0130, R&D systems).

### 2.8. ELISA

The mice were anesthetized after LPS injection and peripheral blood was collected, then subsequently perfused through the heart with 20 mL of cold PBS over a period of 5 min to remove protein from the blood circulation. Mouse brains were harvested and homogenized in cold PBS (1 mL) and centrifuged at 12,000 rpm for 5 min at 4 °C. The concentrations of CXCL1 (P257758, R&D systems) and Tumor Necrosis Factor-alpha (TNF-α) (558534, BD Biosciences) in the brain homogenate supernatant and serum were measured using commercial enzyme-linked immunosorbent assay (ELISA) kits according to the manufacturer’s instructions.

### 2.9. Intravital Microscopy

Intravital microscopy was performed as previously described [15,23]. Briefly, after anesthetization, the right parietal bone was subjected to craniotomy using a high-speed drill. Subsequently, the dura was removed from this site to expose the pial brain vessels. Rhodamine 6G (C6885, Sigma) was injected intravenously (0.5 mg/kg) into the mouse to label the leukocytes. Then, a microscope equipped with a fluorescent light source was used to detect the leukocytes. The data were collected through an sCMOS camera (ORCA-Flash 4.0, HAMAMATSU Photonics, Hamamatsu City, Japan) mounted on the microscope and stored for subsequent analysis. Rolling leukocytes were defined as those cells moving at a slower velocity than the erythrocytes; adherent cells were defined as those that remained stationary for at least 30 s.

### 2.10. Isolation and Culture of Primary Mouse Brain Microvascular Endothelial Cells

Primary cerebral endothelial cells were prepared as previously described [24]. Cortices from 7-week-old C57BL/6J mice were isolated by removing the cerebellum, striatum, optic nerves, and white matter. The outer vessels and the meninges were removed using dry filter paper. Then, the tissue sample was fragmented into small pieces and digested in 10 mL of 0.1% collagen B (C6885, Sigma) supplemented with 30 U/mL DNase I (1002854941, Sigma) for 1.5–2 h at 37 °C with occasional agitation. The suspension was centrifuged at 1000 rpm for 8 min. The resulting homogenate was mixed with 20% BSA (SW3015, Solarbio) in DMEM and centrifuged at 4000 rpm for 20 min at 4 °C. The neural component and the BSA layer were discarded, and the pellet containing the vascular component was further digested in 0.1% collagenase/dispase (10269638001, Roche, Basel, Switzerland) supplemented with 20 U/mL DNase I for 1.5–2 h at 37 °C. The final microvessel pellets were resuspended in DMEM supplemented with 30% FBS (Gibco), 3 ng/mL b-FGF (45033, Peprotech Cranbury, NJ, USA), 10 U/mL heparin, 100 U/mL penicillin, and 100 mg/mL streptomycin. The medium was refreshed every 2 days. The endothelial cells grew to confluency after 7 days. The cells were verified with CD31 staining for endothelial phenotype (Supplementary Figure S1).

### 2.11. Statistical Analysis

All experiments were performed at least three times. Data are presented as means ± SE. Statistical analysis was performed using unpaired Student’s *t*-testing or analysis of variance (ANOVA). For ANOVA, Tukey’s multiple comparisons test was utilized. *p* < 0.05 was considered significant.

## 3. Results

### 3.1. Septic Encephalopathy Induced Increased Expression of CXCR2 in Cerebral Endothelial Cells but Not Neutrophils

**Figure 1:**
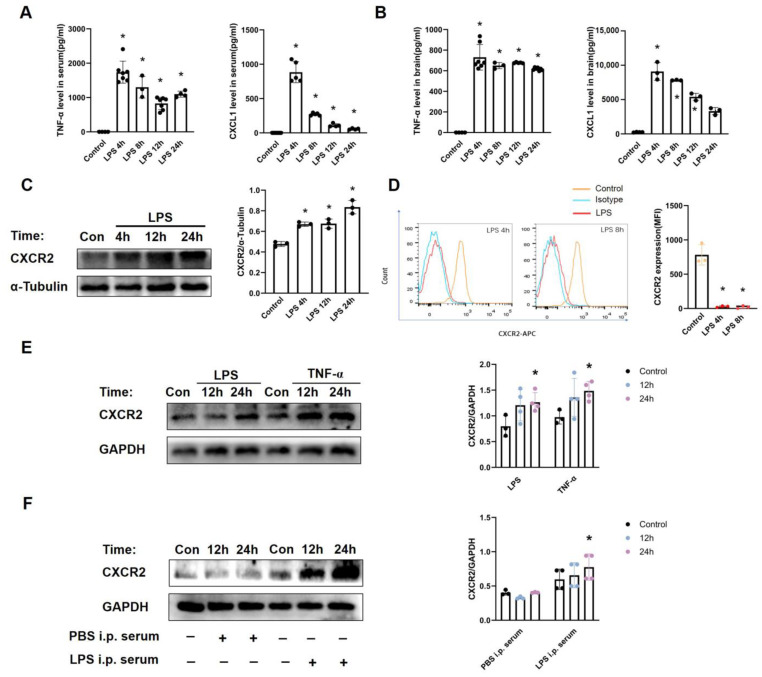
CXCR2 expressions in septic brain, neutrophils, and primary cerebral endothelial cells. (**A**,**B**) Murine SE model was established by LPS injection (10 mg/kg, i.p.). Levels of TNF-α and CXCL1 in the serum and brain tissues at 4 to 24 h after the LPS injection were determined by ELISA (n = 3–7). (**C**) Brain tissues extracted from the mice 4, 12, and 24 h after LPS injection were analyzed by Western blot for expression of CXCR2. Con (control group) mice received PBS injection (n = 3). (**D**) CXCR2 expression on blood neutrophils after LPS or PBS injection was analyzed by FACS (n = 3). (**E**,**F**) Primary cerebral endothelial cells were stimulated by 500 ng/mL LPS, 100 ng/mL TNF-α, or the murine serum after LPS injection (10 mg/kg, i.p.); levels of CXCR2 protein in the primary cerebral endothelial cells were detected by Western blotting (n = 3–4). Con (control group) cells were treated with PBS. The results are presented as the means  ±  SEM and represent a minimum of three mice per group. * *p* < 0.05.

**Figure 2:**
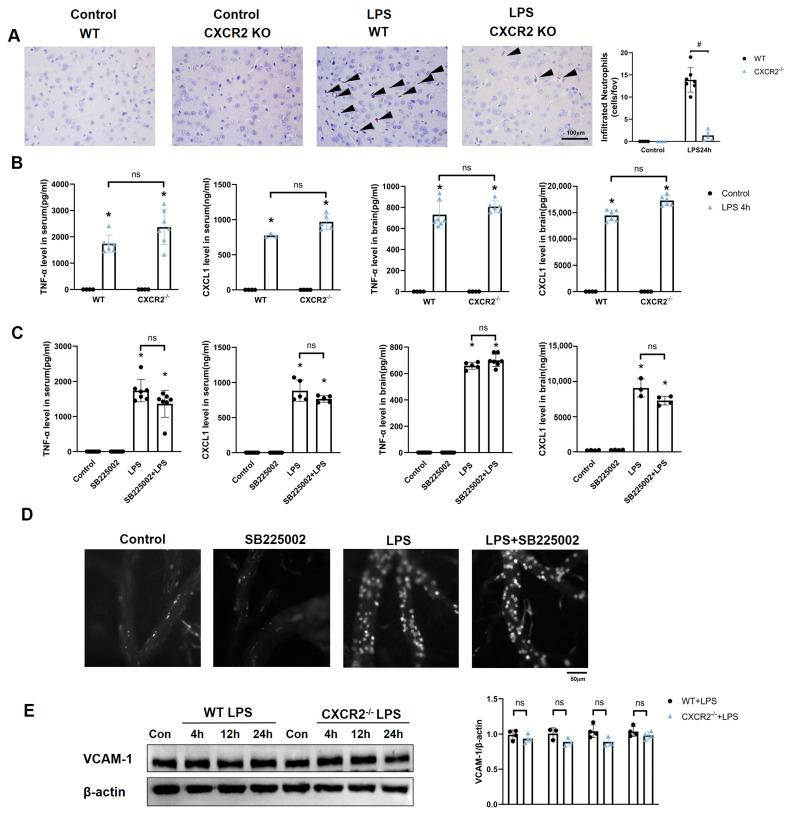
CXCR2 deficiency blocked the infiltrated neutrophils into the brain. (**A**) PBS or LPS was administered to WT mice and CXCR2^−/−^ mice. Esterase staining was used to detect the infiltrated neutrophils into the cortex region of the brain (n = 4–7). Magnification, ×400. Scale bars: 50 μm. Black arrow heads: infiltrated neutrophils. (**B**) The WT and CXCR2^−/−^ mice were treated with LPS (10 mg/kg, i.p.). Levels of TNF-α and CXCL1 in the brain tissue and serum were determined after 4 h by ELISA (n = 3–7). (**C**) Mice received the CXCR2 antagonist SB225002 (10 mg/kg) 0.5 h prior to LPS injection. At 4 h after LPS injection, the levels of TNF-α and CXCL1 in the brain tissue and serum were determined by ELISA (n = 3–8). (**D**) Mice received the CXCR2 antagonist SB225002 (10 mg/kg) 0.5 h prior to LPS injection. At 4 h after i.p. LPS injection, intravital microscopy was performed to show the leukocyte endothelial interactions (rolling flux and leukocyte adhesion) (n = 3). Neutrophils are visualized in the white dots. (**E**) Levels of VCAM-1 in the brain tissue were determined after 4, 12, and 24 h by Western blot after WT and CXCR2^−/−^ mice were injected with LPS (n = 3–4). The results are presented as the means  ±  SEM and represent a minimum of three mice per group. * *p* < 0.05 compared to control groups. #, *p* < 0.05 compared between two selected groups. ns, not significantly different between the two selected groups.

The murine model of SE was established by intraperitoneal injection of high-dose LPS (10 mg/kg i.p.) [25]. After LPS injection, increased levels of TNF-α and CXCL1 (Figure 1A,B) in both the brain and serum confirmed brain and systemic inflammation. And the elevation of infiltrating neutrophils (Figure 2A) into the brain proved the successful establishment of SE. Systemic LPS administration also caused the elevated expression of CXCR2 in the brain (Figure 1C). As CXCR2 signaling is crucial for neutrophil transmigration, we further detected the expression of CXCR2 on neutrophils and cerebral endothelial cells. Interestingly, CXCR2 expression on neutrophils was barely detectable at 4 h and 8 h after LPS injection (Figure 1D). However, inflammatory stimulation of LPS, TNF-α, or serum from SE mice all induced robustly increased expressions of CXCR2 in primary cerebral endothelial cells (Figure 1E,F), which suggests the potential role of cerebral endothelial CXCR2 in SE.

### 3.2. CXCR2 Is Required for Neutrophil Recruitment into the Brain in SE

To further investigate the functional impact of CXCR2 in SE, we detected the neutrophil infiltration and levels of inflammatory cytokines in WT and CXCR2^−/−^ mice after the establishment of SE. CXCR2 deficiency caused remarkedly reduced neutrophil infiltration into the brain cortex (Figure 2A). However, neither CXCR2 deficiency nor CXCR2 inhibitor SB225002 attenuated levels of TNF-α and CXCL1 in the serum and brain tissue of the SE model (Figure 2B,C), which reveals that CXCR2 barely affects the development of the inflammatory microenvironment. Interestingly, although there was almost no neutrophil infiltration observed in the brains of CXCR2^−/−^ mice, CXCR2 inhibitor SB225002 treatment displayed a similar level of neutrophil adhesion to that of control mice in SE (Figure 2B–D). CXCR2^−/−^ mice also displayed a similar level of neutrophil adhesion to that of WT mice [15]. Meanwhile, CXCR2 deficiency does not influence the expression of the adhesion molecule (VCAM-1) in the brain (Figure 2E), suggesting a potential role of brain endothelial CXCR2 in the process of transmigration into the brain during SE, but not in adhesion or rolling, two other critical steps in neutrophil infiltration during septic inflammation.

### 3.3. CXCR2 Deficiency Attenuated SE-Induced BBB Destruction

**Figure 3:**
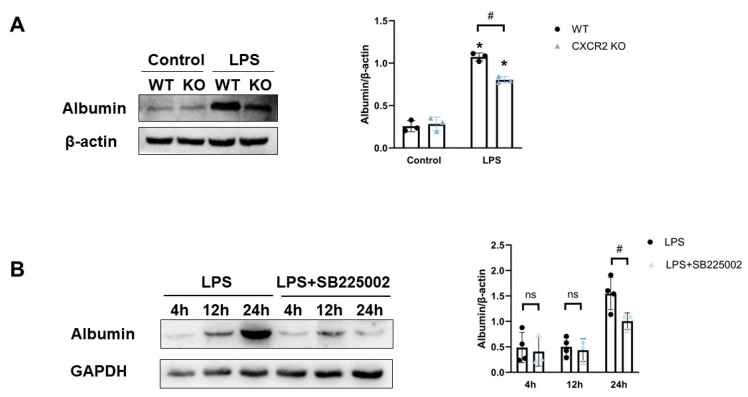
CXCR2 deficiency and inhibition attenuated the LPS-induced destruction of the BBB. (**A**) Ventricular perfusion was used to remove the interference of peripheral circulating proteins after LPS injection for 24 h in WT and CXCR2^−/−^ mice. Then, the levels of albumin were detected to estimate the destruction of BBB permeability. (**B**) Mice received the CXCR2 antagonist SB225002 (10 mg/kg) 0.5 h prior to LPS injection. At 4, 12, and 24 h after LPS injection, levels of albumin were detected to estimate the effect of CXCR2 inhibition on the destruction of BBB permeability. Bar graphs represent means ± SE, n = 3–5. * *p* < 0.05 compared to control groups. #, *p* < 0.05 compared between two selected groups. ns, not significantly different between the two selected groups.

Destruction of the BBB is a key event in the recruitment of neutrophils to the brain. Albumin was detected to evaluate the extent of destruction of the BBB in the murine SE model after ventricular perfusion. CXCR2^−/−^ mice displayed reduced brain albumin levels compared with WT mice (Figure 3A). Furthermore, CXCR2 inhibitor SB225002 treatment similarly attenuated the leakage of albumin into the brain during SE (Figure 3B), revealing that CXCR2 may change the permeability of the BBB by regulating cerebral endothelial integrity, including that of endothelial tight junctions and actin contraction during SE.

### 3.4. CXCR2 Is Essential for CXCL1-Induced Cerebral Endothelial Activation

The neutrophil recruitment cascade is simplified into capture, rolling, adhesion, and (paracellular or transcellular) transmigration. Since the possibility that CXCR2 regulates the process of rolling and adhesion is excluded, CXCR2 is very likely to regulate the transmigration step of neutrophil recruitment. The paracellular pathway of neutrophil transmigration is composed of adhesive junctions between endothelial cells. The actin cytoskeleton is bound to each junction and controls its integrity through actin remodeling. CXCR2-binding chemokine CXCL1 was elevated in serum and brain tissue during SE (Figure 1A,B).

**Figure 4:**
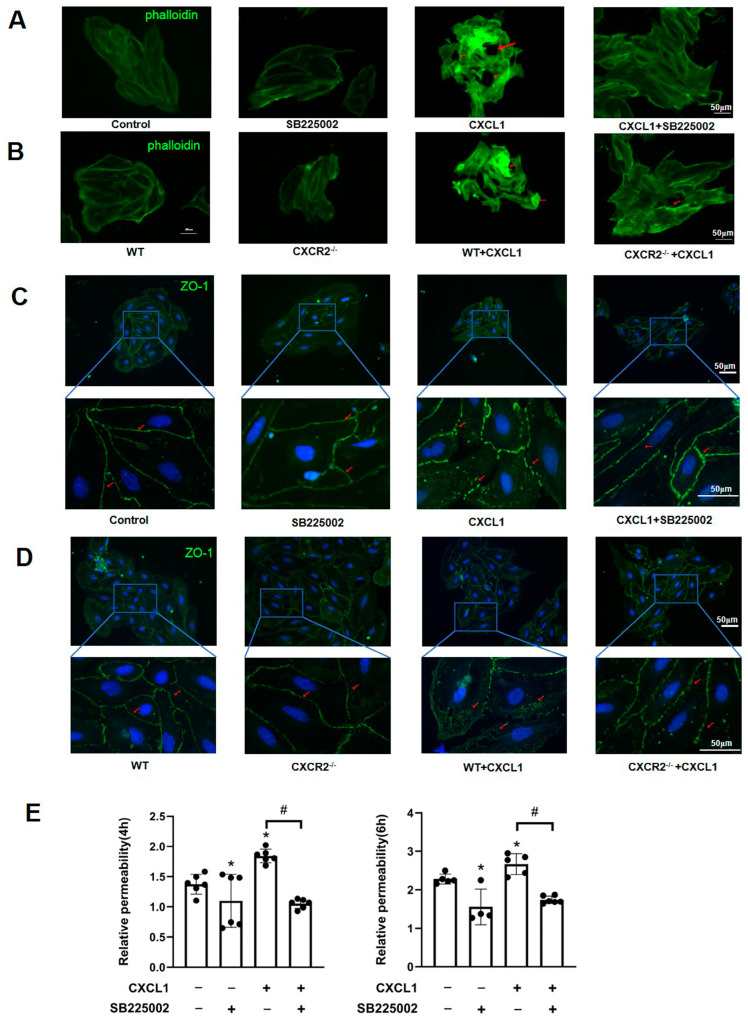
CXCR2 deficiency and inhibition attenuated the F-actin synthesis, ZO-1 downregulation, and endothelial barrier disruption induced by CXCL1 in primary cerebral endothelial cells. (**A**) Primary cerebral endothelial cells from WT mice were pretreated for 30 min with the SB225002 (20 nM) or vehicle only and then treated by CXCL1 (200 ng/mL) or vehicle for 100 min. Immunofluorescent cytochemistry against the F-actin (green) by FITC-phalloidin was assessed to detect the endothelial cell cytoskeleton remodeling (scale bar = 50 µm). (**B**) Primary cerebral endothelial cells from WT or CXCR2^−/−^ mice were stimulated with CXCL1 (200 ng/mL) or vehicle for 100 min. Visualization of F-actin by FITC-conjugated phalloidin was detected by immunofluorescence staining. (**C**,**D**) Immunofluorescence staining against ZO-1 was assessed to detect the distribution of circumferential ZO-1. Red arrows indicate tight junctions, as shown by the periphery ZO-1. (**E**) Endothelial barrier integrity was assessed by transwell assay in primary cerebral endothelial cells treated with SB225002 or CXCL1 for 4 h and 6 h. Representative of at least four independent experiments. *, *p* < 0.05 compared to the control group. #, *p* < 0.05 compared between two selected groups.

To further confirm the mechanisms underlying how CXCR2 regulates cerebral endothelial activation, we next observed the changes in the morphology of the actin cytoskeleton and of tight junction-associated ZO-1 of cerebral endothelial cells in response to CXCL1. CXCL1 stimulation caused a marked redistribution of the subcortical actin network to cytoplasmatic stress fibers and increased gap formation (Figure 4A). However, CXCR2 deficiency and CXCR2 inhibitor SB225002 both markedly blocked the formation of stress fibers and paracellular gaps induced by CXCL1 stimulation (Figure 4A,B). As ZO-1 regulates the size, structure, and stability of endothelial gap junctions, we next examined endothelial ZO-1 distribution upon CXCR2 activation by CXCL1. Simultaneously, we also found that circumferential ZO-1 staining of cerebral endothelial cells was disrupted upon CXCL1 stimulation. In addition, both CXCR2 deficiency and CXCR2 inhibitor SB225002 attenuated the disruption of circumferential ZO-1 (Figure 4C,D). To further confirm the stimulative role of CXCR2 in the disruption of the endothelial barrier, we used an endothelial transwell permeability assay kit to measure the change in endothelial barrier permeability in vitro. Results showed that CXCL1 increased the permeability of the endothelial monolayer with stimulation for 3 h and 4 h, and CXCR2 inhibitor SB225002 partially attenuated the CXCL1-induced increase in endothelial permeability (Figure 4E). Meanwhile, CXCR2 inhibitor SB225002 alone was able to decrease endothelial permeability, suggesting that the function of CXCR2 is required to maintain endothelial permeability under physiological conditions, possibly not dependent on its agonist CXCL1. Together, these results confirmed that CXCR2 promoted neutrophil infiltration into the brain through increasing the formation of stress fibers and gaps between adjacent endothelial cells.

### 3.5. CXCL1-Triggered CXCR2 Activation Induced Cerebral Endothelial Activation through Rho Signaling

**Figure 5:**
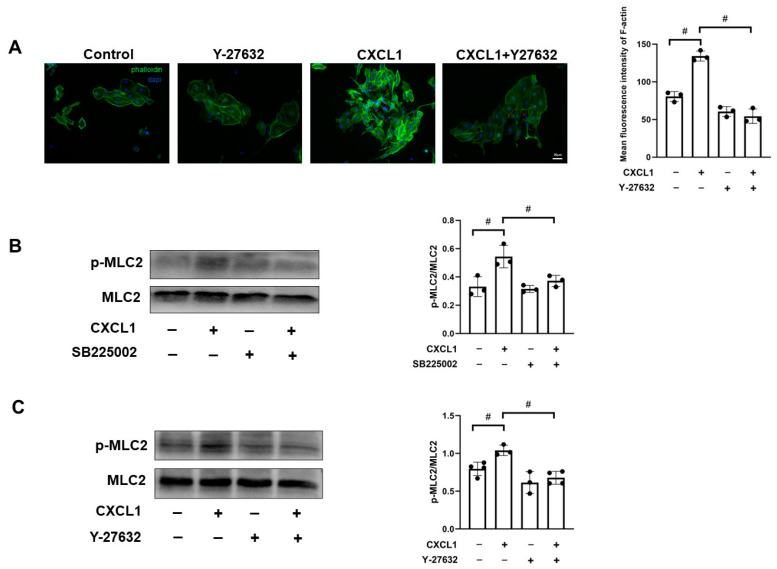
CXCL1-CXCR2 induced endothelial activation through Rho and MLC2 signaling. (**A**) After treatment with Y-27632 (Rho inhibition) or CXCL1, F-actin was stained by FITC-conjugated phalloidin (green), and nuclei was stained by DAPI (blue). Quantification of relative F-actin fluorescence intensity was analyzed. Representations of three independent experiments are shown. (**B**) Primary cerebral endothelial cells from WT mice were pretreated for 30 min with the SB225002 or vehicle only, and then treated by CXCL1 (200 ng/mL) or vehicle for 100 min. Phosphorylation and total MLC2 were measured by Western blot. (**C**) Primary cerebral endothelial cells from WT mice were pretreated for 30 min with the Y-27632 (Rho inhibition) or vehicle only and then treated by CXCL1 (200 ng/mL) or vehicle for 100 min. Phosphorylation and total MLC2 were measured by Western blot. # *p* < 0.05.

G protein-coupled receptor (GPCR)-activated Rho signaling leads to MLC phosphorylation, which next causes actin contraction and opens the tight junctions and adherens junctions between adjacent endothelial cells [26]. Next, we confirmed that CXCR2, as a classic GPCR, when bound to CXCL1, induced cerebral endothelial actin contraction and the subsequent process of neutrophil transmigration in SE. Then, we used Y-27632 (Rho inhibition) to identify whether Rho signaling participates in CXCR2-caused cerebral endothelial activation. Results showed Rho inhibition blocked CXCL1-induced formation of stress fibers and paracellular gaps in cultured brain endothelial cells (Figure 5A). To clarify the signaling pathway, we first examined the effects of CXCR2 inhibitor SB225002 and CXCL1 on MLC2 phosphorylation in CXCL1-induced cerebral endothelial activation. CXCL1 induced obvious MLC2 phosphorylation, and CXCR2 inhibitor SB225002 partially blocked the CXCL1-induced MLC2 phosphorylation in primary cerebral endothelial cells (Figure 5B). In addition, we also confirmed that LPS-induced MLC2 phosphorylation in brain tissues from the murine model of SE was attenuated in CXCR2^−/−^ mice (Supplementary Figure S2). To further confirm whether Rho signaling regulates MLC2 phosphorylation in the downstream signaling pathway in cerebral endothelial activation, we next investigated the effect of Rho inhibition and CXCL1 on MLC2 phosphorylation. Rho inhibition robustly blocked CXCL1-induced MLC2 phosphorylation (Figure 5C). Together, CXCL1 induced CXCR2 activation, which led to MLC2 phosphorylation through Rho signaling. This signaling event subsequently induced cerebral endothelial actin contraction and the final process of neutrophil transmigration in SE.

## 4. Discussion

The current study advances the understanding of the role of CXCR2 in neutrophil infiltration during SE pathogenesis, using cultured brain endothelial cells, CXCR2 knockout mice, and selective CXCR2 inhibitors. The significance of the study lies in multiple levels. Firstly, we confirmed that the levels of CXCR2 and its agonist CXCL1 were increased in brain tissue in a murine model of SE induced by LPS. This elevation was accompanied by elevated CXCR2 expression in cerebral endothelial cells, but not neutrophils, highlighting the potential involvement of cerebral endothelial CXCR2 in SE. Further investigation demonstrated that CXCR2 was essential for neutrophil recruitment into the brain during SE. CXCR2 deficiency markedly reduced neutrophil infiltration, while it did not affect the development of the inflammatory microenvironment. Moreover, CXCR2 deficiency attenuated SE-induced blood–brain barrier destruction, as evidenced by reduced albumin leakage in CXCR2-deficient mice compared to wild-type mice. This suggests a critical effect of CXCR2 on BBB integrity during SE. Additionally, the study elucidated the mechanism by which CXCR2 regulates cerebral endothelial activation. CXCL1 stimulation induced CXCR2-dependent actin cytoskeleton remodeling and the disruption of tight junction-associated ZO-1 in cerebral endothelial cells. CXCR2 inhibition attenuated these effects and maintained endothelial barrier integrity, suggesting a role for CXCR2 in promoting neutrophil infiltration by increasing endothelial permeability. Furthermore, CXCL1-triggered CXCR2 activation was found to induce cerebral endothelial activation through Rho signaling, leading to MLC phosphorylation and subsequent actin contraction, facilitating neutrophil transmigration in SE.

CXCR2 is known to play a pivotal role in orchestrating immune responses within the vasculature, exerting distinct effects on both endothelial cells and neutrophils [27]. On endothelial cells, CXCR2 activation initiates a cascade of events crucial for the regulation of vascular integrity and leukocyte trafficking [15]. Specifically, CXCR2 signaling on endothelial cells promotes the expression of adhesion molecules, facilitating the recruitment and extravasation of neutrophils into inflamed tissues. Moreover, CXCR2 activation induces cytoskeletal rearrangements in endothelial cells, leading to alterations in cell morphology and permeability, which are essential for efficient leukocyte transmigration [15]. Conversely, on neutrophils, CXCR2 activation triggers cellular responses essential for their recruitment and activation at sites of inflammation. Neutrophil CXCR2 signaling enhances cell migration, adhesion to endothelial cells, and the release of inflammatory mediators, thereby amplifying the inflammatory response [28]. Overall, the interplay between CXCR2 on endothelial cells and neutrophils is crucial for the regulation of leukocyte recruitment and vascular inflammation during immune responses. In this study, we confirmed that endothelial CXCR2, but not neutrophil CXCR2, was responsible for neutrophil infiltration in LPS-induced SE. These findings suggest that a brain vascular-targeting therapy on CXCR2 inhibition will be highly effective in reducing brain neutrophilia during SE progression.

Infiltrated neutrophils are considered the major inflammation contributor to SE [3]. Neutrophil infiltration during inflammation involves a series of orchestrated steps essential for their recruitment to sites of tissue injury or infection [29]. Initially, circulating neutrophils are tethered to endothelial cells via selectin-mediated rolling, followed by firm adhesion facilitated by integrin engagement with endothelial adhesion molecules. Subsequently, neutrophils undergo chemotaxis, guided by chemotactic gradients of inflammatory mediators towards the source of infection or tissue damage. This directional migration leads neutrophils to the inflamed tissue. Next, neutrophils crawl along the endothelial surface in a process termed crawling, and then initiate transmigration across the endothelial barrier. The latter is a critical step involving the sequential processes of paracellular or transcellular migration through endothelial junctions and basement membrane penetration. Notably, our findings suggest that CXCR2 selectively exerts a pivotal role, specifically in the process of neutrophil transmigration. Upon the activation by its ligands, including CXCL1, CXCR2 signaling on vascular endothelium triggers intracellular signaling pathways that promote cytoskeletal rearrangements and actin polymerization, opening paracellular gaps, and facilitating the movement of neutrophils through endothelial junctions and basement membranes. This selective action of CXCR2, in response to inflammatory stimuli, exacerbates the inflammatory response and delays the inflammatory resolution. Interestingly, we also notice that CXCR2 inhibitor SB225002 is enough to decrease endothelial permeability (Figure 4E) by itself and partially increase the formation of stress fibers. These findings suggest that the function of CXCR2 is essential for maintaining endothelial permeability in physiological conditions (without LPS challenge), suggesting a complex role of CXCR2 in endothelial integrity homeostasis in disease and health.

Despite the robust findings, this study has some potential limitations. (1) The specificity of the LPS murine model: the study’s findings are based on murine models of SE induced by LPS, which may not fully replicate the complexity of human SE. Differences in immune system dynamics between mice and humans could affect the translatability of the results. While the LPS-SE model is noted for its repeatability and reliability compared to cecal ligation and puncture (CLP) and other models, validation studies should be conducted in other independent models to strengthen the findings. (2) CXCR2 inhibition scope: the study primarily focuses on CXCR2 inhibition without exploring the broader chemokine network. Several key SE-related proinflammatory cytokines [3], such as TNF-α, interleukin-1 beta (IL-1β), interleukin-6 (IL-6), GM-CSF, and interferon-gamma (IFN-γ), are not dependent on CXCR2 signaling and should be explored independently. Future studies should investigate the differential effects of these chemokines and receptors in SE to provide a more comprehensive view of the inflammatory processes. In addition, exploring combination therapies that target multiple pathways involved in neutrophil recruitment and BBB integrity could enhance therapeutic efficacy. Combining CXCR2 inhibitors with other anti-inflammatory agents, such as TNF-α neutralizing antibodies, or neuroprotective agents, might yield better outcomes in managing SE.

CXCR2, a G protein-coupled receptor, plays a significant role in various inflammatory disorders beyond septic encephalopathy. In conditions such as arthritis [30], CXCR2 mediates the recruitment of neutrophils to inflamed joints, contributing to synovial inflammation and joint destruction. In respiratory diseases like chronic obstructive pulmonary disease (COPD) [31] and asthma [32], CXCR2 is crucial for neutrophil chemotaxis to the airways, exacerbating inflammation and tissue damage. Additionally, in ulcerative colitis [33], CXCR2 signaling facilitates neutrophil migration to the gut mucosa, aggravating intestinal inflammation and ulceration. These roles highlight the broad involvement of CXCR2 in mediating inflammatory responses across various tissues. Investigating the role of CXCR2 in the current study will expand the understanding of its potential role, especially in endothelial barrier dysregulation, to facilitate neutrophil infiltration in other inflammatory disorders.

## 5. Conclusions

In conclusion, the findings highlight the critical role of CXCR2 in SE pathogenesis, particularly in neutrophil recruitment, BBB integrity, and cerebral endothelial activation. Targeting CXCR2 signaling may hold therapeutic potential for SE management by mitigating neutrophil-mediated brain injury and preserving BBB integrity.

## Data Availability

The raw data supporting the conclusions of this article will be made available by the authors on request.

## Supplementary Materials

The following supporting information can be downloaded at https://www.mdpi.com/article/10.3390/biomedicines12071536/s1, Figure S1. Identification of primary cerebral endothelial cells with FITC-CD31. Primary cerebral endothelial cells from WT mice were identified by immunofluorescent cytochemistry against CD31 (green) by FITC-CD31, and nuclei was stained by DAPI (blue). Figure S2. CXCR2 knockout decreased phosphorylation of MLC2 in vivo. After LPS injection for 24 h in WT and CXCR2^−/−^ mice, phosphorylation and total MLC2 were measured by Western blot.
