# Supplementary material for: Cerebral Endothelial CXCR2 Promotes Neutrophil Transmigration into Central Nervous System in LPS-Induced Septic Encephalopathy
Source: Biomedicines. 2024 Jul 11;12(7):1536. doi: 10.3390/biomedicines12071536 (PMC11274668; doi:10.3390/biomedicines12071536)
Supplement: Supplementary file 1 [file biomedicines-12-01536-s001.zip › Suppementary figures.pptx]

## Slide 1
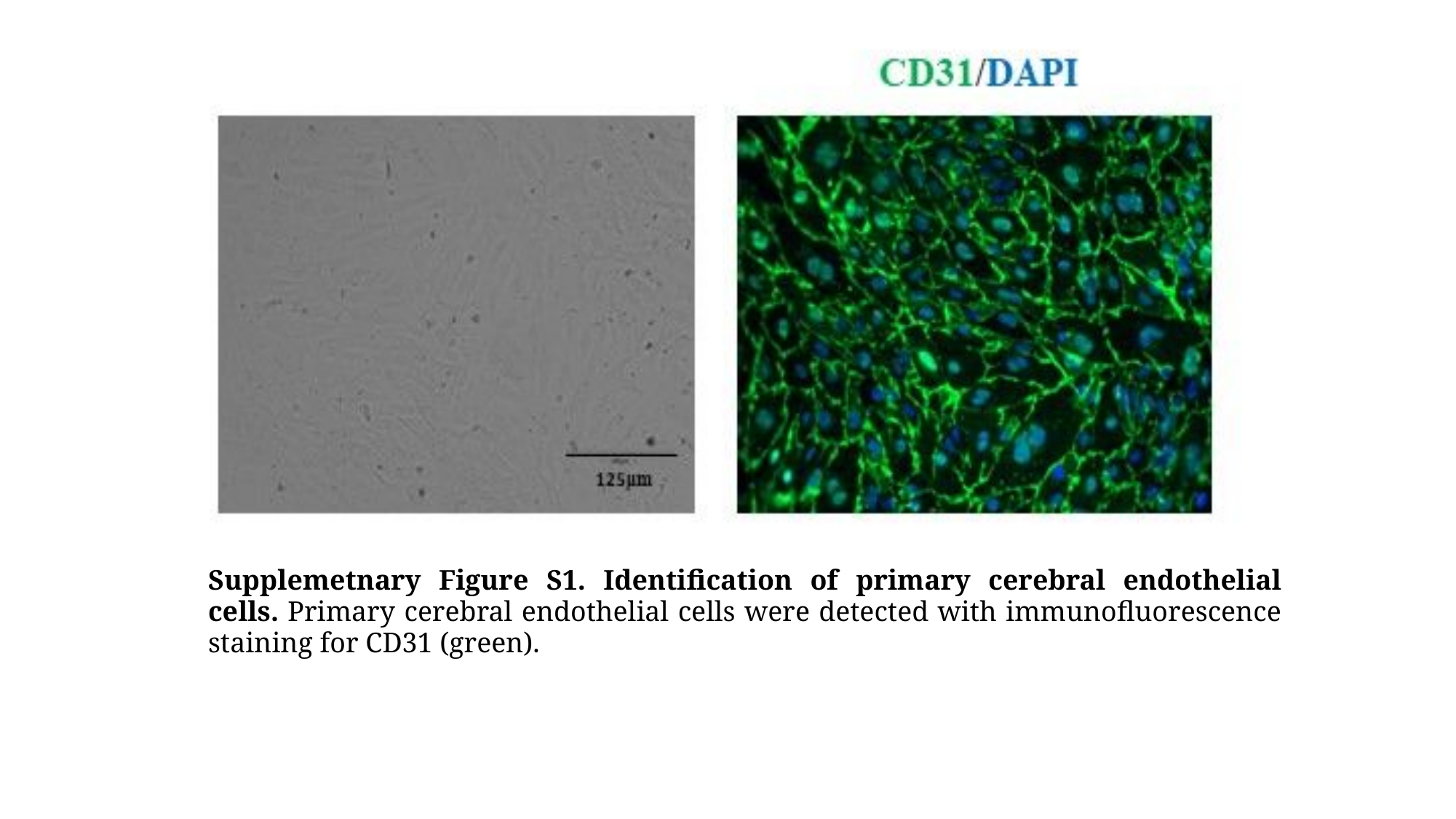

Supplemetnary Figure S1. Identification of primary cerebral endothelial cells. Primary cerebral endothelial cells were detected with immunofluorescence staining for CD31 (green).

## Slide 2
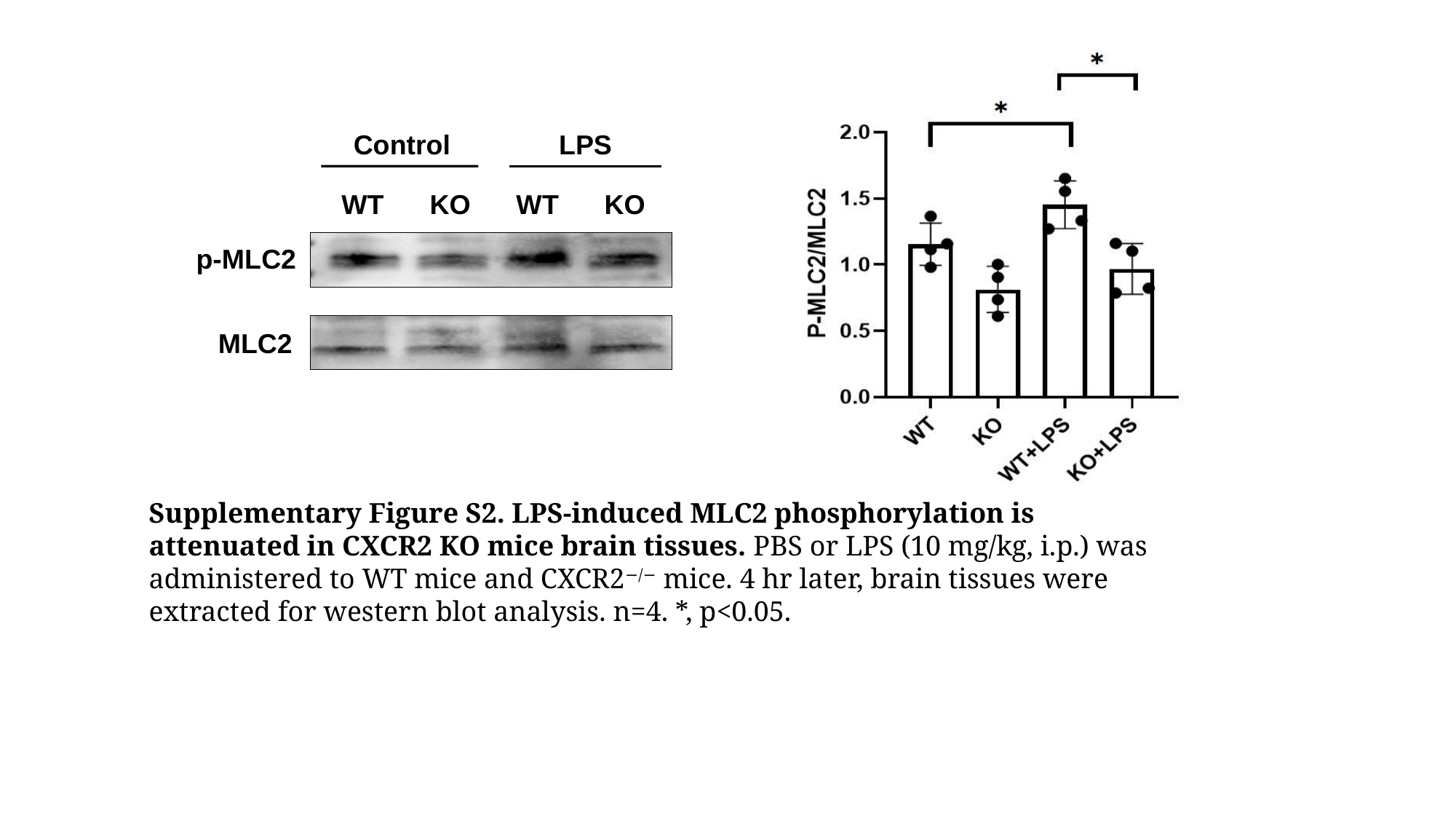

Control
LPS
 WT KO WT KO
p-MLC2
MLC2
Supplementary Figure S2. LPS-induced MLC2 phosphorylation is attenuated in CXCR2 KO mice brain tissues. PBS or LPS (10 mg/kg, i.p.) was administered to WT mice and CXCR2−/− mice. 4 hr later, brain tissues were extracted for western blot analysis. n=4. *, p<0.05.
